# The Potential Role of *Citrus limon* Powder as a Natural Feed Supplement to Boost the Productive Performance, Antioxidant Status, and Blood Biochemistry of Growing Rabbits

**DOI:** 10.3390/ani9070426

**Published:** 2019-07-07

**Authors:** Hamada A. M. Elwan, Dawood Hosni Dawood, Sally Mohamed Abd El-Aziz El-Shafei, Atef Abd El-Mohsen Abd El-Rahman, Shaker A. Abdel-Latif, Mohamed Mohany, Faleh Alqahtani, Saeed Alqahtani, Salim S. Al-Rejaie

**Affiliations:** 1 Animal and Poultry Production Department, Faculty of Agriculture, Minia University, El-Minya 61519, Egypt; 2 Agricultural Chemistry Department, Faculty of Agriculture, Mansoura University, Mansoura 35516, Egypt; 3 Department of Agricultural Chemistry, Faculty of Agriculture, Minia University, El-Minya 61519, Egypt; 4 Department of Pharmacology and Toxicology, College of Pharmacy, King Saud University, Riyadh 11451, Saudi Arabia

**Keywords:** *Citrus limon*, oxidative biomarkers, GC-MS analysis, rabbits

## Abstract

**Simple Summary:**

Although some studies have suggested that the flavonoids in *Citrus limon* possess numerous biological functions, including antioxidant, anti-inflammatory, antiallergic, antiviral, antiproliferative, antimutagenic, and anticarcinogenic activities, a rabbit model has not been used to establish the beneficial effects and possible antioxidant activity of dried *C. limon*. Therefore, we investigated the hemato-biochemical alteration, thyroid activity, and antioxidant responses of dried *C. limon* as a powder in growing rabbits. By employing New Zealand White rabbits, we demonstrated that rabbits fed a diet supplemented with 1% dry lemon powder (DLP) or 2% DLP had an increase in their productive performance, better feed conversion, enhanced hematological and biochemical values, improved antioxidant enzyme activity, and inhibited lipid peroxidation. These results suggest that dry lemon supplementation might play a role as a growth enhancer for rabbits when administered at a maximum of 2% in the rabbit diet. In general, the addition of *Citrus limon* improved growth performance, physiology, and antioxidant status in serum and liver of the growing rabbit.

**Abstract:**

The current study examined the influence of *Citrus limon* (dry lemon) on the hemato-biochemical profiles, and antioxidant indices of growing rabbits. Forty-eight growing New Zealand White rabbits (age, eight weeks; weight, 1543.33 ± 25 g) were allocated into three groups (16 animals each), the first group was (control) fed a basal diet, whereas the second and third groups were supplemented with dried lemon, 1% or 2% DLP, respectively. A GC-MS analysis of more than 27 active constituents was performed. Feed conversion efficiency was (*p* < 0.05) better with diets containing 1% or 2% dry lemon, compared to the control group. Hematological indexes were increased significantly with the addition of DLP compared to those in the control group. Adding 1% or 2% dry lemon to rabbit diet increased (*p* < 0.05) enzymatic and non-enzymatic antioxidant activities (TAC, SOD, GSH, GST, and CAT) in serum and liver tissues. Taken together, these data reveal the advantages and antioxidant effects of dry lemon supplementation for growing rabbits once supplemented at a maximum of 2% in their daily diet.

## 1. Introduction

Nutritional factors play an important role in the protection against the deleterious consequences of free radicals. Natural antioxidants-rich diets can significantly affect the enhancement of reactive antioxidants and reduce the risk of some diseases induced by free radicals generation. A high enough level of antioxidants in a diet is considered to encourage immunological processes and correctly elevate the defensive abilities of the cell [1].

Over the years, plants have served as precious sources of bioactive compounds for humans and animals, thereby possessing great potential for use in the discovery of new drugs [2]. *Citrus* fruit has the highest antioxidant activity [3] as it contains many flavonoids, vitamin C, and carotenoids [4]. There are numerous bioflavonoids in *Citrus limon* such as hesperidin, rutin, and naringin, which exhibit many pharmacological actions in the body [5]. Citrus fruits, especially *C. limon*, have been scientifically proven to display many health benefits as well as limiting the risk of diseases due to their richness in vitamins and minerals and other active constituents [6].

Numerous studies have proposed that *C. limon* may display antifungal [7] and anti-cancer [8] activities. Also, some studies have suggested that its flavonoids possess numerous biological functions, including anti-inflammatory, antioxidant, antiviral, antiallergic, antiproliferative, antimutagenic, and antitumor activities [9,10,11,12]. Essential oils of *C. limon* has also been found to prevent damage to the brain, lung, and intestine induced by aspirin toxicity [13].

Several progenies of farm rabbits were known to produce a higher amount of meat at a rate greater than 10–15 times of their weight [14]. Rabbit meat is preferred for its nutritional value compared with other meat products because of its low calorie content and high digestion [15]. Proper care to maintain the physiological status of rabbits is an essential factor in the production of high-quality products that meat consumers expect. Nobakht [16] demonstrated that different concentrations of dried lemon pulp in diets had marked influence on broilers performance. Azra et al. [17] found that consuming lime peel or fresh lime juice induced the highest changes in serum antioxidant capacity. Flavonoids in *Citrus* was found to protect against hyperglycemia via increasing liver glycolysis and lowering liver gluconeogenesis [18]. Hesperidin and naringin, both *C. limon* active compounds, were also found to significantly lowered the blood glucose level [19]. Moreover, dietary dried citrus pulp supplementation may improve laying hens performance by their positive effects on lipid profile and blood glucose levels [20].

Previous studies demonstrated the use of citrus pulp, juice, and peel meal as an animal feed additive without any accompanying side effects [21]. In addition, dietary fresh or dry fruit of *C. limon* was demonstrated to contribute to the maintenance of optimum health due to its health-promoting phytochemicals. To date, however, impactful investigations on dried *C. limon* have not been performed to establish its beneficial effect and possible antioxidant activity with a rabbit model. Hence, this experiment was employed to explore the hemato-biochemical profiles, thyroid activity, and antioxidant responses of dried *C. limon* as a powder in growing rabbits.

## 2. Materials and Methods

This study was in accordance with the guidelines of Egyptian Research Ethics Committee, Animal and Poultry Production Department, Faculty of Agriculture, Minia University, and approved the guidelines contained in the Guide for the Care and Use of Laboratory Animals (2011). Project identification MU-FA-APPD/1/M/321/2018.

### 2.1. In Vitro Studies on Dry Lemon Powder (DLP)

#### 2.1.1. Preparation of Dry Lemon

Lemon was obtained from a private commercial market in El-Minya Governorate, Egypt and dried at 40 °C until constant weight. After the drying process, the obtained dried lemon was ground, sieved (1 mm mesh) and finally stored in air-tight dark polyethylene bags at 25 °C. The composite sample of the lemon powder was placed in a plastic sample bag for chemical and nutritional analysis.

#### 2.1.2. Preparation and Extraction of Methanol Extract

One hundred g of DLP was macerated with methanol at 25 °C for 24 h, then was filtered using a filter paper (Whatman No. 1), the residual underwent an extraction process twice by the same manner. Methanol was removed at 40 °C in a rotary evaporator. To obtain the methanol extract, the samples were dried in desiccators [22].

#### 2.1.3. Total Phenolic Compounds Content

Total phenolic compounds content was measured by the Folin-Ciocalteu method with little modifications [23]. In brief, 50 μL of the extract was mixed with 3 mL of distilled water, 250 μL of Folin-Ciocalteu reagent, and 750 μL of 7.5% Na_2_CO_3_, which was then vigorously shaken for two minutes and incubated for 30 min at 25 °C. Absorbance values were estimated at 760 nm (UV-VIS Spectrophotometer, Shimadzu, Kyoto, Japan). Quantification of total phenolic compounds in the extracts was obtained by gallic acid standard curve. Results are expressed as mg gallic acid equivalent/g dried sample. All measurements were done in triplicate.

#### 2.1.4. Assessment of Total Flavonoid Content

Total flavonoid content was measured by the aluminum chloride colorimetric assay method [24]. In brief, 0.5 mL of the extract was added to 1.5 mL of absolute ethanol, 0.1 mL of 10% aluminum chloride, 0.1 mL of 1 M potassium acetate, and 3 mL of distilled water. The yield mixture was mixed by vortex, incubated at 25 °C for 30 min; the absorbance was measured at 415 nm (UV-VIS Spectrophotometer, Shimadzu). The concentration of total flavonoid content in the methanolic extract was calculated by quercetin standard curve. Data are presented as mg quercetin equivalent/g dried sample.

#### 2.1.5. Determination of Antioxidant Activity

Antioxidant activity was determined using 2, 2-biphenyl 1-picrylhydrazyl (DPPH) as reported by Oliveira et al. [25]. In brief, 100 μL of methanolic extract was mixed with 3.9 mL DPPH (0.004%) methanolic solution. Absorbance was measured at 517 nm after incubation at 25 °C in the dark for 60 min. The reference compound was ascorbic acid. The following formula was used to estimate % inhibition:

Inhibition (%) = (A control − A test)/A control × 100(1)

where; A control = the absorbance of the control reaction and A test = the absorbance of the sample (methanol extract).

#### 2.1.6. Gas Chromatography/Mass Spectrometry (GC/MS) Analysis

Mass spectra of chemical compounds were conducted using GC/MS (Agilent 6890 (19091S-433) series GC-MS) equipped with an HP-5MS column (diameter length 30 m; diameter 0.25 mm; film thickness 0.25 μm) and mass spectrometer programmed to operate at temperature of 30 °C—280/300 °C, withhold time of 5 min, and rate of 10 °C/min. The conditions of chromatography were injection mode, column flow rate (1 mL/min), split; and carrier gas, helium (purity 99.999%). Mass library search (NIST based AMDIS software) with their relative retention indices was used to identify GC-MS spectra compounds [26].

#### 2.1.7. Nutritional Analysis of Rabbit Diets and Citrus Limon

Using A.O.A.C., standard methods, dry matter (DM), crude fiber (CF), crude protein (CP) and ether extract (EE) were determined [27]. By difference, nitrogen-free extract (NFE) was calculated. According to the method of Van Soest et al. [28], fiber fractions of neutral detergent fiber (NDF), acid detergent fiber (ADF) and acid detergent lignin (ADL) were assessed. Whereas, hemicellulose was calculated by the difference between NDF and ADF and cellulose by the difference between ADF and ADL. Analysis of amino acid in the basal diet and DLP was performed by hydrolyzing the samples by HCl (6 M) at 110 °C for 24 h using HPLC (Hitachi L-8900 Amino Acid Analyzer, Tokyo, Japan; Table 1).

### 2.2. In Vivo Studies on DLP

#### 2.2.1. Experimental Animals

The experiments were conducted at the faculty farm (Animal and Poultry Production Department, Faculty of Agriculture, Minia University). The rabbits were housed individually in galvanized wire cages (40 × 50 × 35 cm) that were provided with feeders and an automatic drinking water system. They were housed in a semi-closed building and maintained under the same managerial, hygienic and environmental conditions. A period of 14–16 h of daylight was also provided.

#### 2.2.2. Rabbits Ration and Dry Lemon Supplementation

Experimental rations of the dry lemon were prepared in the form of pellets (0.3 cm) and mixed with other feed ingredients for each batch at 0, 1 or 2% for control, and 1% DLP and 2% DLP, respectively.

#### 2.2.3. Animals Grouping and Treatments

Forty-eight growing New Zealand White (NZW) rabbits (1 Male:1 Female), aged 8 weeks, (average weight, 1543.33 ± 25 g) were allocated by weight into three groups (16 animals each); the first group was fed a basal diet free of dry lemon (control), while the second and third groups recieved the same basal diet containing 1% or 2% DLP, respectively, as a supplement for 8 weeks. Body weight and feed intake were recorded every week.

#### 2.2.4. Physiological Response

At the end of the experiment, rabbits (n = 8 per group) were fasted for 12 h, weighed and handily slaughtered. Then, the slaughtered animals were de-skinned, dressed out, and their carcass weight was recorded. Also, weights of edible offal’s (liver, heart, and kidneys) were recorded.

#### 2.2.5. Hematological Parameters

At the end of the experiment (16 weeks), blood was collected from the marginal ear vein in clean tubes with or without anticoagulant substance. Coagulated samples were centrifuged at 2515.5× *g* for 15 min to separate the serum, which was stored at −80 °C until analysis. Whole blood was analyzed after sampling for physical blood characteristics using a veterinary hematology analyzer (VetScan HM5 Hematology System Abaxis Europe, UK).

#### 2.2.6. Serum Total Antioxidant Capacity and Lipid Profiles

Serum total antioxidant capacity was determined, according to Trachootham et al. [30]. Concentrations of total cholesterol (TC) and triacylglycerols were measured after hydrolysis, followed by oxidation of the sample. HDL-cholesterol levels were assessed using the methods of Lopes-Virella et al. [31]. Serum LDL-c concentration was calculated using Friedewald equation: LDL (mg/dL) = TC − HDL − (TG/5) which was modified by Ahmadi et al. [32] as:

LDL (mg/dL) = TC/1.19 + TG/1.9 − HDL/1.1 − 38(2)

The estimated very low-density lipoprotein cholesterol (VLDL-c), was obtained by the equation of Friedewald as follows:

VLDL-c = Triacylglyceroles/5(3)

#### 2.2.7. Thyroid Hormones

The levels of triiodothyronine (T3) and thyroxin (T4) were determined by an ELISA technique (Boston, MA, USA).

#### 2.2.8. Serum and Hepatic Oxidative Stress Biomarkers

Serum and liver tissue concentrations of superoxide dismutase (SOD), catalase (CAT), glutathione-S-transferase (GST), glutathione peroxidase (GSH-Px) and malondialdehyde (MDA) were evaluated by kits (BioMed chemical company, Cairo, Egypt).

### 2.3. Statistical Analysis

The experiment was carried out with a completely randomized design (CRD) of three treatments and 16 replicates per group. The obtained results were analyzed with the statistical analysis system software (SAS, Version 9.1.3, 2003, Cary, NV, USA) using the generalized linear model (GLM) procedure [33]. Significant differences among treatment means for each trait in the experiment were measured using Duncan’s multiple range test. The following mathematical model was used:

Xij = µ + Ti + Eij(4)

where; Xij = value observed in each experimental unit, µ = mean population, Ti = the effect of each treatment, and Eij = the effect of experimental errors.

## 3. Results

### 3.1. Dry Lemon In Vitro Studies

#### 3.1.1. Determination of Chemical Profile, Total Phenolics, Total Flavonoids Content, and Total Antioxidant Capacity of Dry Lemon

The chemical profile of dry lemon based on DM basis was tabulated in Table 1; 9.40% CP, 12.62% CF, 4.93% EE, 62.02% NFE, 4.52% ash, 60.58% NDF, 37.48% ADF, 5.64% ADL. Temporarily, the hemicellulose and cellulose were 23.10% and 31.84%, respectively. Also, amino acids content in DL powder were analyzed after hydrolyzing using HPLC Amino Acid Analyzer (Table 1). The respective total phenolics, total flavonoids, and total antioxidant capacity in the methanolic extract were 120.57 mg/g gallic acid equivalent, 31.28 mg/g quercetin equivalent, and 49.12% based on the percentage of DPPH radicals inhibition.

#### 3.1.2. GC-MS Analysis

**Figure 1:**
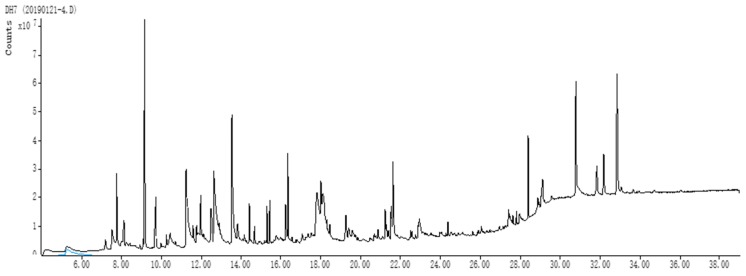
GC-MS analysis of the methanolic extract of dry lemon powder.

GC-MS was used to analyze dry lemon extract major metabolites. More than 27 phytochemicals (Figure 1, Table 2) were present in the methanolic extract of dried lemon after separation based on retention times. The chemical constituents obtained were characterized by different classes of compounds viz: alkaloids, phenols, terpenes, glycosides, coumarins, fatty acids, and phytosterols. Based on the percentage area, the analysis demonstrated the existence of 5-(hydroxymethyl)-2-(methoxymethyl)furan (24.49%), imidazole (18.3%), d-limonene (8.40%), β-methoxy-(s)-2-furanethanol (8.20%), β-d-glucopyranoside, methyl (7.10%), 5,7-dimethoxy-3-nitro-2H-1-benzopyran-2-one (5.39%), quinic acid (4.66%), 6-methoxychroman-2-one (2.84%), scoparone (2.81%), and squalene (2.61%) as the major phytochemicals. Table 3 displays the phytochemicals identified in the lemon methanol extract along with their formula and compound nature.

### 3.2. In Vivo Study of DLP

#### 3.2.1. Effect of Dry Lemon Dietary Levels on Productive Performance of Growing Rabbits

The effect of dietary treatments on the growth performance of growing rabbits is shown in Table 3. Final weight, weight gain, and average daily gain were significantly (*p* < 0.05) increased as affected by dietary dry lemon levels compared with the control group. Animals fed on control diet (0% DLP) recorded the highest feed conversion ratio (*p* < 0.05) compared to other groups. 

#### 3.2.2. The Influences of Dietary Treatments on Growing Rabbit Carcass Traits

Rabbits fed 1% or 2% DLP had the heavier (*p* < 0.05) carcass weight compared with the control group; however, there was no significant difference between the two treatments. Values for liver weight, liver percentage, kidney weight, and kidney percentage were significantly (*p* < 0.01) decreased when rabbits were fed 1% and 2% DLP compared with the control group. Moreover, rabbits supplemented with 1% DLP did not have any significant (*p* > 0.05) changes in liver percentage, kidney weight, and kidney percentage in comparison with that fed 2% DLP. Also, significant differences were not found, except for body weight, among all groups, especially for heart weight and heart % (Table 4).

#### 3.2.3. Rabbit Physiological Changes after Dried Lemon Supplementation

(1) Hematological Alterations

As shown in Table 5, the results indicated that rabbits fed on 2% DLP had the higher (*p* < 0.05) RBC’s count, PCV, Hb, MCV, and MCH compared to the other dietary treatments; however, there were no significant (*p* > 0.05) differences between 1% or 2% DLP on MCH. Instead, a significant decrease in MCHC value (*p* < 0.05) was observed when rabbits were fed on 2% DLP compared to that in those fed to other groups. Rabbits fed on 1% DLP did not display any significant (*p* > 0.05) differences in RBC’s count and MCV compared to those fed the control diet. In addition, they had the highest MCHC value for all groups. Also, there were no significant differences (*p* > 0.05) among all groups on platelets number (PLT) and its indices (MPV, PCT, PDW-cv, and PDW-SD).

(2) Total Antioxidant Capacity and Lipid Profiles

The effect of dietary treatments on triacylglycerol, cholesterol, HDL-c, LDL-c, VLDL-c, levels is shown in Table 6. Rabbits fed a diet containing a high amount of dried lemon supplementation (2% DLP) had the highest values (*p* < 0.05) for HDL-c, and TAC compared to others fed diets containing the lowest level DLP (1%), and the control diet. Also, rabbits fed on the control diet had the highest (*p* < 0.05) triacylglycerol, cholesterol, and LDL-c levels compared to those fed supplemented diets.

(3) Thyroid Hormones as Affected by Dry Lemon Supplementation

Table 7 shows that DLP supplementation in the diet of growing rabbits significantly (*p* < 0.05) increased their T3 level by 20.26 and 26.92% (1% and 2% DLP, respectively) compared to those fed the control diet, respectively. There is no significant (*p* > 0.05) between DLP levels on the T3. The concentrations of T3 were 0.8343 and 0.8805 (ng/mL) for 1% and 2% DLP, consecutively. Thyroxin, T4 levels significantly (*p* < 0.05) increased by 14.33% and 19.05% for rabbits fed on DLP (1% or 2%) in comparison with those fed the control diet, respectively. While the T4 level was insignificantly (*p* > 0.05) greater for rabbits fed diet contains 2% DLP when compared to those fed 1% DLP. Also, T4/T3 ratio significantly (*p* < 0.01) increased when rabbits fed on 2% DLP were compared with the control group. At the same time, results indicated that the (T4/T3 ratio) did not significantly (*p* > 0.05) differ between rabbits fed on diets containing 1% or 2% DLP levels.

(4) Serum and Hepatic Oxidative Stress Biomarkers as Affected by Dry Lemon Supplementation

The levels of serum oxidative stress biomarkers (Table 8) such as GST, CAT, SOD, GSH, and GSH-Px were significantly (*p* < 0.05) increased when 1% or 2% DLP was administrated to growing rabbits compared to those of the control group. Whereas GST activity in rabbit serum significantly (*p* < 0.05) increased by 3.89% and 5.09% compared to that in the control group, a significant difference was not found between treated groups 1 or 2% DLP. Additionally, data indicated that the catalase activity (CAT) did not significantly differ with 1% or 2% DLP treated levels. In contrast, lipid peroxidation, MDA level, significantly (*p* < 0.05) decreased when rabbits fed on DLP (1% or 2%) were compared with the control group, as shown in Table 8. Moreover, the highest level of 2% DLP had the lowest MDA values compared with other groups. As shown in Table 8, the results revealed that the values of SOD, GSH and GSH-Px activity of treated rabbits 1% or 2% DLP, increased significantly (*p* < 0.05) compared to the levels for those fed the control diet. Additionally, hepatic oxidative biomarkers displayed the same trend in serum.

## 4. Discussion

Dietary antioxidant supplements can enhance the physiological and productive statuses of animals by modifying metabolic processes. The current study aimed to investigate the influences of dry lemon on the hemato-biochemical alterations, and antioxidant indices of growing rabbits. Firstly, we sought to investigate in vitro phytochemical compounds of dry lemon (total phenolics, total flavonoids content) as well as total antioxidant capacity. The presence of phenolic compounds and flavonoids in the diet play an important role in cancer chemoprevention. Additionally, the health benefits of phenolic compounds depend on their consumption and bioavailability [34]. Phenolic compounds have a wide range of biological and chemical activities, including radical scavenging properties. Total phenolic content and total flavonoids content were 120.57 mg/g and 31.28 mg/g, respectively. In contrast, Sultana et al. [35] reported respective values of 158.79 mg/g and 29.33 mg/g. The differences between these findings and our data may be attributed to several factors including the regions and seasons of the sample collection, sections (peels, fruits), used for analysis, and the extraction procedure.

Secondly, the DPPH method was employed to assess the antioxidant activity of the dried lemon methanolic extract.

Through phytochemical analysis, the percentage of DPPH radicals inhibition of *C. limon* extract was found to be 49.12%. A similar, antioxidant activity (43.60%) of lemon peel was reported by Reyhan et al. [36] and may have been due to its high phenolic content. Our results also demonstrated that *C. limon* extracts possess a high concentration of total polyphenols (120.57 mg/g) and flavonoids (31.28 mg/g).

The antioxidant activity of lemon fruits might be due to the occurrence of ascorbic acid content and biologically active secondary metabolites, which have been known to display significant antioxidant activity [37]. Data of the present study showed that dry lemon methanolic extract contains marked content of total phenolics, flavonoids, and antioxidant activity. Several phytochemical molecules were identified by dry lemon extract GC analysis.

Generally, terpenoids, glycosides, and coumarins give the dry lemon extract their antioxidant and antimicrobial properties [38]. The significance and activities of these molecules attribute to their lipophilic character of hydrocarbon skeleton and functional groups [39]. GC analysis revealed that dry lemon methanolic extract had a relatively high content of 5-(Hydroxymethyl)-2-(dimethoxymethyl) furan, imidazole, and limonene, which are known to possess crucial antioxidant activity. Interactions among the components of the dry lemon methanolic extract could also affect their activity because of the synergistic effect of their main constituents [40]. Increasing terpenes levels makes essential oils more effective [41]. A substantial number of studies reported that phenolic compounds not only cause the antioxidant activity, but that all the phytochemical molecules are responsible for this activity [42].

Data revealed that adding both levels of DLP (1% or 2%) to the diet of growing rabbit diets significantly increased their body weight, weight gain, and carcass weight. The improvement of rabbit performance by adding DLP to their diets may be due to the use of lemon as whole fruit, which led to an increase in the appetite and a higher consumption of feed. Also, lemon fruits were enriched with the most essential amino acids, potent antioxidant activity, total phenolic and total flavonoids compounds including anthocyanins, coumarins, flavonoids, flavones, isoflavones, catechins, isocatechins, and lignans, which might be represent the optimum antioxidant activity of dry lemons. A similar study conducted by Chaudry et al. [43] revealed that adding 5% of citrus pulp into broiler diets did not have any significant effects on feed consumption. This has been confirmed in the present study. Furthermore, our results concerning feed conversion differed from those of Mourao et al. [44] who found that boilers fed 5% or 10% citrus pulp had significantly higher FCR than the control birds. However, Wang et al. [21] added 0%, 4%, 8%, 12%, or 16% DCP to a geese diet until 70 d of age, and they found that geese that were fed on diets containing 4% DCP resulted in higher average daily gain than other groups. Additionally, diets containing 16% DCP led to an elevation in the average daily feed intake and a higher FCR compared to control and 12%, respectively. Diet of geese supplement with DCP showed no effects on carcass traits across the treatment groups [45]. However, rabbits fed the control diet had the highest values for the absolute and proportions of the liver, kidney, and heart compared to treated groups. The highest values of carcass and dressing percentages were observed when rabbits were fed the higher amount of DLP (2%) than the lower amount (1% DLP). No effect was detected in the absolute and proportion of the kidney based on addition amount. The previous results disagree with the study conducted by Petracci et al. [15], which showed that gizzard weight and abdominal fat percentages were significantly affected by the addition of DLP in broiler diets. However, numerically, DLP had beneficial effects on some carcass traits.

Following DLP supplementation, a significant improvement in all hematological parameters was found. Moreover, 2% DLP led to the highest values for RBCs, PCV, Hb, MCV, and MCH (5.96%, 8.57%, 8.56%, 2.46% and 2.41%, respectively) compared to the control group. The enhancement in most hematological parameters might be due to the presence of many active compounds such as vitamin C, flavonoids, iron, and pyridoxine. Azra et al. [17] reported similar results, as they found that different *C. limon* doses (0.2, 0.4, and 0.6 mL/kg) could significantly affect different hematological parameters in healthy rabbits.

The results of the current study showed that *Citrus limon* significantly increased the thyroid hormone levels (T3, T4, and T3/T4 ratio) compared to those found for the control group. This finding is supported by Miler et al. [45], who found that *Citrus* flavanones increased serum TSH without changing T4 levels. Moreover, by supplementing rabbit diets with 1% or 2% DLP showed a significant reduction in serum triglycerides, cholesterol, LDL-c, and VLDL-c were significantly reduced, while the level of HDL-c level statistically increased compared to the level in control animals. The hypolipidemic effect of *C. limon* may be due to its flavonoids content, which can reduce blood cholesterol, triglycerides, and LDL-c levels based on its antioxidant effect [46]. Complete methoxyylation of ring A with Citrus flavonoids seems to be an optimal structural change that exhibits substantial effects on the modulation of hepatic lipid metabolism via the suppressed secretion of lipoproteins with apoB in HepG2 cells [47]. Other optimal molecular structures, such as tangeretin and nobiletin, exhibited a lower effect on blood cholesterol levels and triglyceride concentration. Also, *Citrus* flavonoids, such as hesperidin and naringin content lacking a fully methoxylated ring, can virtually exhibit little or no reduction in the effects of lipids in humans [48].

In the current study, a significant elevation in the levels of serum HDL was detected [49], suggesting that *C. limon* may serve as a valuable supplement in rabbit diet. Other compounds, such as pectin, a component of the plant cell wall, was reported to play a role in reducing cholesterol. This is due to the structure of flavonoids containing numerous OH groups, which can supply H atoms to quench free radicals, ultimately contributing to its potent antioxidant property [50].

Three major levels of defense mechanisms were found in the antioxidant system of living cells. The activities of SOD, GSH-PX, and CAT is the first, which act to prevent free radical formation [51]. The second level act by chain-breaking antioxidants (vitamin E, C, carotenoids) that disrupt chain multiplication and formation. The third level restores or eliminates damaged molecules from the cell by the help of specific enzymes [52].

More importantly, this study showed that the activities of antioxidant enzymes such as GST CAT, SOD, and GSH were increased, while that of MDA was markedly decreased in the rabbits of the DLP treatment group compared to those in the control group. The increase in activities of antioxidant enzymes may be related to active DLP constituents such as ascorbic acid, flavonoids, and phenolic compounds and agree with many previous studies [53,54,55], where citrus extracts, such as citrus peel, *C. karna*, and *C. limetta* were demonstrated to potentially have antioxidant bioactivity. The most important defense mechanism of dry lemon phenolic compounds is based on the absorption and neutralization of free radicals [56]. However, previous reports have placed more emphasis on the antioxidant capacity of *C. limon* [4,57,58]. *C. limon* also contains several constituents such as 3-carene, imidazole, D-limonene, geraniol, and squalene as well as other compounds reported as potent antioxidants that can prevent microsomal lipid peroxide and protein degradation of ROS [59,60,61,62,63], These data are in accordance with our phytochemical study results which highlighted *C. limon* as a powerful scavenging agent.

## 5. Conclusions

The present study demonstrated that rabbits fed a diet supplemented with 1% DLP or 2% DLP had an increase in their productive performance, better feed conversion, enhanced hematological and biochemical values, improved antioxidant enzyme activity, and inhibited lipid peroxidation. These results suggest that DLP would be effective in improving their physiological status and antioxidant capacity during growth.

## Figures and Tables

**Table 1:** Ingredients, amino acids and nutritional analysis of rations and dry lemon.

Elements	Dry Lemon *	Experimental Groups		
		Control	1% DLP	2% DLP
Yellow corn (%)	-	44.00	44.00	44.00
Wheat bran (%)	-	40.50	40.50	40.50
Soybean mail (44% crude protein) (%)	-	13.50	13.50	13.50
Lime (%)	-	0.5	0.5	0.5
Sodium chloride (%)	-	1.00	1.00	1.00
Vitamin and mineral premix^**^ (%)	-	0.50	0.50	0.50
Dry lemon powder (%)	-	0	1	2
Analyzed nutritional composition (%)				
Moisture	6.51	5.92	5.90	5.91
OM	88.97	89.20	89.36	89.17
CP	9.40	18.65	18.86	18.87
CF	12.62	10.42	10.83	10.78
EE	4.93	2.94	2.95	3.01
NFE	62.02	57.19	56.72	56.51
Ash	4.52	4.88	4.74	4.92
NDF	60.58	41.45	42.02	42.69
ADF	37.48	24.25	26.09	25.17
ADL	5.64	5.44	5.51	5.81
Hemicellulose	23.10	17.20	15.93	17.52
Cellulose	31.84	18.81	20.98	19.36
Calculated gross energy, kcal/g	4.10	4.16	4.17	4.17
Analyzed amino acids (g/100 g)				
Alanine	0.746	0.849	0.868	0.876
Arginine	0.365	0.827	0.842	0.847
Aspartic acid	0.265	0.780	0.794	0.798
Cysteine	1.032	0.250	0.272	0.283
Glutamic acid	0.465	0.644	0.660	0.665
Glycine	0.323	0.600	0.615	0.619
Histidine	0.265	0.370	0.384	0.388
Isoleucine	0.142	0.628	0.641	0.644
Leucine	0.365	1.162	1.177	1.182
Lysine	0.266	0.672	0.686	0.689
Methionine	0.365	0.389	0.404	0.409
Phenylalanine	0.613	0.616	0.634	0.641
Proline	0.511	0.679	0.696	0.702
Serine	0.323	0.683	0.698	0.702
Threonine	0.412	0.611	0.626	0.632

* Percentage of dry weight content of fresh lemon fruit peels = 24.05 ± 0.52; Gross energy was calculated according to NRC [29], where g CP = 5.72 kcal, EE = 9.50 kcal CF = 4.79 kcal and NFE = 4.03. SEM = standard error of means; DM = dry matter; OM = organic matter; CP = crude protein; CF = crude fiber; EE = ether extract; NFE = nitrogen free extract; NDF = neutral detergent fiber; ADF = acid detergent fiber; ADL = acid detergent lignin.

**Table 2:** Phytochemical active constituents of dried lemon methanolic extract.

Retention Time (min)	Compound Name	Mol. Formula	Compound Nature	Peak Area (%)
7.198	3-Carene	C_10_H_16_	Bicyclic monoterpenes	1.13
7.530	Imidazole	C_3_H_4_N_2_	Alkaloids	18.3
7.769	β-methoxy-(s)-2-furanethanol	C_7_H_10_O_3_	Glycosides	8.20
9.166	d-Limonene	C_10_H_16_	Monoterpenes	8.40
11.312	3-Methyl-4-propyl-2,5-furandione	C_8_H_10_O_3_	Glycosides	0.43
12.899	Geraniol	C_10_H_18_O	Monoterpenes	1.79
13.540	5-(Hydroxymethyl)-2-(dimethoxymethyl)furan	C_8_H_12_O_4_	Glycosides	24.49
13.828	2-Methoxy-5-vinylphenol	C_9_H_10_O_2_	Phenols	1.82
14.420	Geranyl Acetate	C_12_H_20_O_2_	Monoterpenes	1.66
15.304	Caryophyllene	C_15_H_24_	Sesquiterpenes	0.44
15.487	2,6-Di-Tert-butylphenol	C_14_H_22_O	Phenols	0.14
16.259	β-d-Glucopyranose, 1,6-anhydro-	C_6_H_10_O_5_	Glycosides	0.53
16.348	β-Bisabolene	C_15_H_24_	Sesquiterpenes	1.59
17.809	Quinic acid	C_7_H_12_O_6_	Organic acids	4.66
18.005	6-Methoxychroman-2-one	C_10_H_10_O_3_	Coumarins	2.84
18.115	β-d-Glucopyranoside, methyl	C_7_H_14_O_6_	Glycosides	7.10
19.260	Methyl 3-(4-hydroxy-3-methoxyphenyl) propanoate	C_11_H_14_O_4_	Phenols	1.89
21.229	Hexadecanoic acid	C_16_H_32_O_2_	Saturated Fatty acid	0.38
21.523	Scoparone	C_11_H_10_O_4_	Coumarins	2.81
21.618	5,7-Dimethoxy-3-nitro-2*H*-1-benzopyran-2-one	C_11_H_9_O_6_	Coumarins	5.39
22.944	Cis-13-Octadecenoic acid	C_18_H_34_O_2_	Long-chain Fatty acids	0.13
27.469	Imperatorin	C_16_H_14_O_4_	Coumarins	1.34
28.400	Squalene	C_30_H_50_	Triterpenes	2.61
29.128	Geranyl linolenate	C_28_H_48_O_2_	Triterpenes	0.08
31.846	Campesterol	C_28_H_48_O	Phytosterol	0.43
32.185	Stigmasterol	C_29_H_48_O	Phytosterol	0.21
32.855	γ-Sitosterol	C_29_H_50_O	Phytosterol	1.22

**Table 3:** The effects of dried lemon supplementation on productive performance of growing rabbits.

Parameters	Experimental Groups			*p*-Value
	Control	1% DLP	2% DLP	
Initial weight, g	1518.33 ± 8.46	1548.33 ± 8.30	1563.33 ± 8.33	0.11
Final weight, g	2567.66 ± 6.49 ^b^	2959.00 ± 5.44 ^a^	2966.33 ± 5.42 ^a^	<0.01
Body weight gain, g	1049.33 ± 6.25 ^b^	1396.33 ± 6.12 ^a^	1418 ± 6.15 ^a^	<0.01
Average daily gain, g	18.73 ± 0.04 ^b^	24.93 ± 0.02 ^a^	25.32 ± 0.03 ^a^	0.04
Feed intake g/day	123 ± 1.89	126 ± 2.01	130 ± 2.02	0.06
Feed conversion ratio (g feed/g gain)	6.56 ± 0.09 ^a^	5.05 ± 0.05 ^b^	5.13 ± 0.05 ^b^	0.03

^a,b^ Values within a row with different letters differ significantly (*p* < 0.05). Control = Lemon free diet; 1% DLP = 1 % dry lemon powder; 2% DLP = 2% dry lemon powder.

**Table 4:** Carcass traits of growing rabbits after dietary dried lemon supplementation.

Parameters	Experimental Groups			*p*-Value
	Control	1% DLP	2% DLP	
Carcass Weight (including head) (g)	1194.50 ± 17.50 ^b^	1798.00 ± 24.74 ^a^	1819.00 ± 24.10 ^a^	<0.01
Carcass %	46.45 ± 0.61 ^c^	61.26 ± 0.59 ^b^	63.20 ± 0.51 ^a^	<0.01
Dressing %	49.29 ± 0.53 ^c^	63.53 ± 0.49 ^b^	65.44 ± 0.50 ^a^	<0.01
Liver weight (g)	54.20 ± 6.30 ^a^	51.0 ± 6.90 ^b^	47.90 ± 8.90 ^c^	<0.01
Liver %	2.10 ± 0.11 ^a^	1.73 ± 0.10 ^b^	1.66 ± 0.11 ^b^	<0.01
Kidney Weight (g)	13.15 ± 3.05 ^a^	10.52 ± 4.31 ^b^	11.00 ± 4.29 ^b^	<0.01
Kidney %	0.51 ± 0.05 ^a^	0.36 ± 0.05 ^b^	0.38 ± 0.05 ^b^	<0.01
Heart Weight (g)	5.40 ± 0.10	5.24 ± 0.11	5.50 ± 0.10	0.66
Heart %	0.20 ± 0.02	0.18 ± 0.02	0.19 ± 0.02	0.32

^a,b,c^ Values within a row with different letters differ significantly (*p* < 0.05). Control = Lemon free diet; 1% DLP = 1% dry lemon powder; 2% DLP = 2% dry lemon powder.

**Table 5:** The effects of dried lemon supplementation of different hematological parameters.

Parameters	Experimental Groups			*p*-Value
	Control	1% DLP	2% DLP	
RBC’s (10^6^/µL)	5.87 ± 0.02 ^b^	5.89 ± 0.02 ^b^	6.22 ± 0.02 ^a^	0.03
PCV (%)	35.00 ± 0.02 ^b^	34.00 ± 0.02 ^c^	38.00 ± 0.02 ^a^	0.02
Hb (g/dL)	11.67 ± 0.02 ^c^	11.98 ± 0.02 ^b^	12.67 ± 0.02 ^a^	0.02
MCV (fL)	59.62 ± 1.55 ^b^	57.72 ± 1.57 ^b^	61.09 ± 1.56 ^a^	0.02
MCH (pg)	19.88 ± 0.13 ^b^	20.33 ± 0.12 ^a^	20.36 ± 0.15 ^a^	0.04
MCHC (g/dL)	33.34 ± 0.01 ^b^	35.23 ± 0.01 ^a^	33.34 ± 0.01 ^b^	0.02
RDW-cv (%)	16.6 ± 0.11	16.5 ± 0.10	16.54 ± 0.10	0.06
RDW-SD (fL)	41.2 ± 1.58	40.98 ± 1.57	41.02 ± 1.59	0.07
PLT (10^3^/µL)	170 ± 1.55	169.9 ± 1.57	170.01 ± 1.55	0.06
MPV (fL)	6.99 ± 0.34	6.78 ± 0.36	6.89 ± 0.33	0.06
PCT (%)	0.11 ± 0.01	0.10 ± 0.01	0.10 ± 0.01	0.06
PDW-cv (%)	29.35 ± 0.54	29.25 ± 0.58	29.45 ± 0.56	0.07
PDW-SD (fL)	6.89 ± 0.47	6.80 ± 0.46	6.92 ± 0.44	0.06

^a,b,c^ Values within a row with different letters differ significantly (*p* < 0.05). RBC’s = red blood cells; PCV = peaked cell volume; Hb = hemoglobin; MCV = mean corpuscular volume; MCH = mean corpuscular hemoglobin; MCHC= mean corpuscular hemoglobin concentration; RDW-cv = coefficient of variation of red blood cell distribution width; RDW-SD = standard deviation of red blood cell distribution width; PLT= platelets; MPV = mean platelet volume; PCT = procalcitonin; PDW-cv = coefficient of variation of platelet distribution width; PDW-SD = standard deviation of platelet distribution width. Control = lemon free diet; 1% DLP = 1 % dry lemon powder; 2% DLP = 2% dry lemon powder.

**Table 6:** Changes in serum levels of lipid profiles and total antioxidant capacity in rabbits treated with dietary dried lemon.

Parameters	Experimental Groups			*p*-Value
	Control	1% DLP	2% DLP	
Triacylglycerol (mg/dL)	181.17 ± 1.22 ^a^	179.01 ± 1.24 ^b^	171.60 ± 1.23 ^c^	0.04
Cholesterol (mg/dL)	238.53 ± 1.44 ^a^	184.40 ± 1.46 ^b^	166.97 ± 1.48 ^c^	0.02
HDL-Cholesterol (mg/dL)	94.97 ± 0.47 ^b^	95.62 ± 0.48 ^b^	117.51 ± 0.45 ^a^	0.04
LDL-Cholesterol (mg/dL)	171.46 ± 1.05 ^a^	120.34 ± 1.09 ^b^	89.69 ± 1.07 ^c^	0.03
VLDL-Cholesterol (mg/dL)	36.23 ± 0.26 ^a^	35.80 ± 0.24 ^b^	34.32 ± 0.22 ^c^	0.02
TAC (mM/L)	68.02 ± 0.02 ^c^	69.02 ± 0.02 ^b^	70.25 ± 0.02 ^a^	0.02

^a,b,c^ Values within a row with different letters differ significantly (*p* < 0.05). HDL-Cholesterol = high-density lipoprotein cholesterol; LDL-Cholesterol = low-density lipoprotein cholesterol; VLDL-Cholesterol = very low-density lipoprotein cholesterol; TAC = total antioxidant capacity. Control = lemon free diet; 1% DLP = 1 % dry lemon powder; 2% DLP = 2% dry lemon powder.

**Table 7:** Effects of dried lemon diet on thyroid hormones of growing rabbits.

Items	Experimental Groups			*p*-Value
	Control	1% DLP	2% DLP	
Triiodothyronine (ng/mL)	0.6937 ± 0.02 ^b^	0.8343 ± 0.02 ^a^	0.8805 ± 0.02 ^a^	0.02
Thyroxin (µg/dL)	11.44 ± 1.66 ^b^	13.08 ± 1.68 ^a^	13.62 ± 1.65 ^a^	0.02
Triiodothyronine/Thyroxin ratio	0.0607 ± 0.01 ^b^	0.0637 ± 0.01 ^ab^	0.0647 ± 0.01 ^a^	0.02

^a,b,c^ Values within a row with different letters differ significantly (*p* < 0.05). Control = lemon free diet; 1% DLP = 1 % dry lemon powder; 2% DLP = 2% dry lemon powder.

**Table 8:** Effects of dried lemon diet on serum and hepatic oxidative stress biomarkers of growing rabbits.

Items	Experimental Groups			*p*-Value
	Control	1% DLP	2% DLP	
Serum				
GST (U/L)	67.58 ± 3.04 ^b^	70.21 ± 3.06 ^a^	71.02 ± 3.04 ^a^	0.02
CAT (U/L)	20.21 ± 1.15 ^b^	22.01 ± 1.16 ^a^	22.10 ± 1.14 ^a^	0.03
SOD (U/mL)	1.90 ± 0.05 ^c^	2.98 ± 0.05 ^b^	3.25 ± 0.05 ^a^	0.02
GSH (mg/dL)	0.65 ± 0.01 ^c^	0.72 ± 0.01 ^b^	0.78 ± 0.01 ^a^	0.02
GSH-Px activity (mU/mL)	11.37 ± 1.30 ^b^	13.38 ± 1.26 ^a^	13.35 ± 1.28 ^a^	0.02
MDA (nmol/ mL)	2.90 ± 0.20 ^a^	2.41 ± 0.18 ^b^	2.25 ± 0.16 ^c^	0.02
Liver				
GST (U/g tissue)	123.02 ± 4.05 ^b^	135.56 ± 4.04 ^a^	136.58 ± 4.07 ^a^	<0.01
CAT (U/g tissue)	30.12 ± 2.24 ^c^	37.29 ± 2.26 ^b^	41.00 ± 2.22 ^a^	0.03
SOD (U/mg tissue)	134.20 ± 3.56 ^c^	150.29 ± 3.58 ^b^	158.57 ± 3.54 ^a^	<0.01
GSH (mg/g tissue)	11.23 ± 0.58 ^b^	12.96 ± 0.54 ^a^	13.31 ± 0.56 ^a^	0.02
GSH-Px activity (U/g tissue)	142.73 ± 3.16 ^c^	155.83 ± 3.17 ^b^	160.53 ± 3.13 ^a^	0.02
MDA (nmol/g protein)	11.22 ± 1.80 ^a^	7.13 ± 1.78 ^b^	6.18 ± 1.79 ^b^	0.02

^a,b,c^ Values within a row with different letters differ significantly (*p* < 0.05). GST = glutathione S transferase; CAT = catalase; SOD = superoxide dismutase; GSH = glutathione; GSH-Px = glutathione peroxidase. Control = lemon free diet; 1% DLP = 1 % dry lemon powder; 2% DLP = 2% dry lemon powder.
